# Características sociodemográficas associadas ao baixo peso e ao excesso de peso em adultos com 50 anos ou mais (ELSI-Brasil): diferenças entre sexos 

**DOI:** 10.1590/0102-311XPT037023

**Published:** 2024-02-02

**Authors:** Rantiele Bruna Machado Martins, Juliana Lustosa Torres, Bruno de Souza Moreira, Maria Fernanda Lima-Costa, Nair Tavares Milhem Ygnatios

**Affiliations:** 1 Centro Universitário Santa Rita, Conselheiro Lafaiete, Brasil.; 2 Núcleo de Estudos em Saúde Pública e Envelhecimento, Fundação Oswaldo Cruz/Universidade Federal de Minas Gerais, Belo Horizonte, Brasil.; 3 Departamento de Medicina Preventiva e Social, Universidade Federal de Minas Gerais, Belo Horizonte, Brasil.; 4 Instituto René Rachou, Fundação Oswaldo Cruz, Belo Horizonte, Brasil.

**Keywords:** Antropometria, Fatores Sociodemográficos, Idoso, Índice de Massa Corporal, Sobrepeso, Anthropometry, Sociodemographic Factors, Aged, Body Mass Index, Overweight, Antropometría, Factores Sociodemográficos, Anciano, Índice de Masa Corporal, Sobrepeso

## Abstract

Os objetivos foram descrever a prevalência de baixo peso e excesso de peso, avaliados pelo índice de massa corporal (IMC), estratificada por sexo e faixa etária, e analisar as características sociodemográficas associadas ao IMC em mulheres e homens mais velhos. Trata-se de uma análise transversal de 8.974 participantes com ≥ 50 anos da linha de base do *Estudo Longitudinal da Saúde dos Idosos Brasileiros* (ELSI-Brasil, 2015-16). O IMC foi classificado em baixo peso, eutrofia e excesso de peso de acordo com a idade do participante. Foi utilizado modelo de regressão logística multinominal, considerando-se as características sociodemográficas de mulheres e homens. Os resultados evidenciaram maior prevalência de excesso de peso nas mulheres em comparação aos homens (64,1% *vs.* 57,3%). Em ambos os sexos, a prevalência de baixo peso foi maior nos mais longevos, enquanto que o excesso de peso foi menor. Nas mulheres, a chance de baixo peso foi maior do que a chance de eutrofia naquelas solteiras/viúvas/divorciadas (OR = 1,95; IC95%: 1,42-2,66) e nas residentes na área rural (OR = 1,58; IC95%: 1,01-2,49), ao passo que a chance de excesso de peso foi menor do que a chance de eutrofia nas residentes na área rural (OR = 0,78; IC95%: 0,62-0,97) e em todas as macrorregiões geográficas relativas à Região Sul. Para os homens, a chance de excesso de peso foi menor do que a chance de eutrofia entre solteiros/viúvos/divorciados (OR = 0,58; IC95%: 0,48-0,69). Os mais ricos apresentaram menor chance de baixo peso (OR = 0,59; IC95%: 0,38-0,90), bem como maior chance de excesso de peso (OR = 1,52; IC95%: 1,20-1,92). Em conclusão, as características sociodemográficas associadas ao IMC diferiram entre os sexos.

## Abstract (en)

The objective were to describe the prevalence of underweight and overweight, assessed by body mass index (BMI), stratified by sex and age group, and to analyze the sociodemographic characteristics associated with BMI in older women and men. This is a cross-sectional analysis of 8,974 participants aged ≥ 50 years from the baseline of the *Brazilian Longitudinal Study of Aging* (ELSI-Brasil, 2015-2016). BMI was classified as underweight, eutrophy, and overweight according to the participant’s age. A multinomial logistic regression model was used, considering the sociodemographic characteristics of women and men. The results showed a higher prevalence of overweight in women compared to men (64.1% vs. 57.3%). In both sexes, the prevalence of underweight was higher in the longest-lived individuals, while overweight was lower. In women, the chance of underweight was higher than the chance of eutrophy in those who were single/widowed/divorced (OR = 1.95; 95%CI: 1.42-2.66) and in those living in rural areas (OR = 1.58; 95%CI: 1.01-2.49), while the chance of being overweight was lower than the chance of being eutrophy in those living in rural areas (OR = 0.78; 95%CI: 0.62-0.97) and in all geographic macro-regions related to the South Region. For men, the chance of being overweight was lower than the chance of being eutrophy among single/widowed/divorced individuals (OR = 0.58; 95%CI: 0.48-0.69). The richest had a lower chance of being underweight (OR = 0.59; 95%CI: 0.38-0.90), as well as a higher chance of being overweight (OR = 1.52; 95%CI: 1.20-1.92). In conclusion, the sociodemographic characteristics associated with BMI differed between the sexes.

## Abstract (es)

Los objetivos fueron describir la prevalencia de bajo peso y sobrepeso, evaluados a través del índice de masa corporal (IMC), estratificada por sexo y grupo de edad, y analizar las características sociodemográficas asociadas al IMC en mujeres y hombres mayores. Se trata de un análisis transversal de 8.974 participantes con ≥ 50 años de la línea de base del *Estudio Longitudinal Brasileño sobre el Envejecimiento* (ELSI-Brasil, 2015-2016). Se clasificó el IMC en bajo peso, eutrofia y sobrepeso conforme la edad del participante. Se utilizó el modelo de regresión logística multinomial, teniendo en cuenta las características sociodemográficas de mujeres y hombres. Los resultados evidenciaron una prevalencia más alta de sobrepeso en las mujeres en comparación con los hombres (64,1% *vs.* 57,3%). En ambos sexos, la prevalencia de bajo peso fue más alta en los grupos de mayor edad, mientras que la prevalencia del sobrepeso fue menor. La chance de bajo peso fue más alta que la chance de eutrofia en las mujeres solteras/viudas/divorciadas (OR = 1,95; IC95%: 1,42-2,66) y en las que viven en el área rural (OR = 1,58; IC95%: 1,01-2,49), mientras que la chance de sobrepeso fue menor que la chance de eutrofia en las que viven en el área rural (OR = 0,78; IC95%: 0,62-0,97) y en todas las macrorregiones geográficas relacionadas a la región Sur. La chance de sobrepeso fue menor que la chance de eutrofia entre los hombres solteros/viudos/divorciados (OR = 0,58; IC95%: 0,48-0,69). Los más ricos presentaron una chance menor de bajo peso (OR = 0,59; IC95%: 0,38-0,90), así como una chance más alta de sobrepeso (OR = 1,52; IC95%: 1,20-1,92). En conclusión, las características sociodemográficas asociadas al IMC difirieron entre los sexos.

## Introdução

A má nutrição, em todas as suas formas (por exemplo, baixo peso, sobrepeso e obesidade), constitui um dos principais problemas de saúde pública da atualidade, afetando todos os países do mundo ^1^. É resultado dos processos das transições epidemiológica, demográfica e nutricional, as quais contribuíram para mudanças significativas nos ambientes alimentares e na qualidade da dieta mundial, e foram exacerbadas pela globalização, em especial nos países de baixa e média renda ^2^^,^^3^^,^^4^.

Nas últimas décadas, essas transições estão relacionadas à mudança de uma predominância do baixo peso para elevadas prevalências de excesso de peso, comumente classificados nos estudos epidemiológicos por meio do índice de massa corporal (IMC) ^5^. No Brasil, de 2006 a 2020, houve um aumento de 14,9% do excesso de peso na população com 18 anos ou mais, segundo dados da pesquisa *Vigilância de Fatores de Risco e Proteção para Doenças Crônicas por Inquérito Telefônico* (Vigitel) ^6^^,^^7^. Atualmente, cerca de 54% dos homens e metade das mulheres nessa faixa etária apresentam excesso de peso. Essa prevalência tende a aumentar com o avançar da idade em ambos os sexos ^7^. Por exemplo, mulheres e homens mais velhos (50-59 anos) apresentaram prevalência de excesso de peso em torno de 62% e 65%, respectivamente, frente a aproximadamente 38% e 43% das mulheres e homens mais jovens (20-29 anos), sendo maior entre os homens mais escolarizados, mas menor nas mulheres mais escolarizadas ^8^.

Por outro lado, a prevalência de baixo peso é relativamente baixa, em torno de 2%, na população adulta de 20 a 59 anos ^8^. Ainda de acordo com dados da Vigitel, em 2019, 12% dos idosos (60 anos ou mais) e 15% das idosas brasileiras apresentaram baixo peso, cerca de seis vezes maior em comparação com a população adulta ^8^, o que pode ser devido à perda de massa muscular inerente ao envelhecimento ^9^. Essa prevalência era maior em 2009, sendo de 20% nos idosos e 18% nas idosas, e demonstrou relação inversa com idade, escolaridade e renda mensal *per capita*^10^. Além disso, o baixo peso era mais prevalente em idosos que se autodeclararam pretos e amarelos, moravam sozinhos, residiam na área rural e nas regiões Nordeste e Centro-oeste. Já o excesso de peso foi mais elevado na área urbana e nas regiões Sul e Sudeste ^10^.

Embora os determinantes do peso sejam muito complexos, envolvendo uma interação entre fatores biológicos, comportamentais, ambientais e sociais, os resultados supracitados refletem as influências das disparidades sociodemográficas, condicionantes das desigualdades sociais e econômicas do país, no IMC de sua população ^11^.

A maioria dos estudos com representatividade nacional tem dado mais atenção à avaliação do excesso de peso na população em geral ^12^^,^^13^. No entanto, faltam estudos representativos para adultos mais velhos brasileiros. Os impactos econômico, social e de saúde do baixo peso, assim como do excesso de peso, são graves e duradouros, tanto para os indivíduos, quanto para a sociedade. Portanto, a temática configura-se como um desafio em saúde pública e requer mais investigações, principalmente no Brasil, cuja transição nutricional ocorre atrelada a um contexto de desigualdades sociais, econômicas e de saúde ^14^.

Nesse contexto, este estudo teve como objetivos: (i) descrever a prevalência de desvios nutricionais do IMC (baixo peso e excesso de peso), estratificada por sexo e faixa etária, em adultos mais velhos brasileiros; e (ii) analisar as características sociodemográficas associadas ao IMC em mulheres e homens mais velhos. Os resultados poderão contribuir para melhor compreensão das diferenças demográficas e sociais do país nesse indicador antropométrico, para subsidiar programas e políticas públicas direcionadas ao enfrentamento da má nutrição.

## Métodos

### Fonte de dados e desenho do estudo

Para esta análise, foram utilizados dados da linha de base do *Estudo Longitudinal da Saúde dos Idosos Brasileiros* (ELSI-Brasil), cuja amostra foi delineada para representar a população brasileira com 50 anos ou mais, residentes em 70 municípios das cinco macrorregiões geográficas do país. A linha de base foi conduzida entre 2015 e 2016, utilizando um delineamento com seleção amostral por estágios, que combinou a estratificação dos municípios como unidade primária, seguidos dos setores censitários e dos domicílios. Todos os residentes de 50 anos ou mais dos domicílios selecionados foram elegíveis para a entrevista individual, totalizando 9.412 participantes. A descrição metodológica detalhada pode ser consultada em outras publicações ^15^^,^^16^.

O ELSI-Brasil é coordenado pelo Instituto René Rachou, Fundação Oswaldo Cruz (Fiocruz Minas), e pela Universidade Federal de Minas Gerais (UFMG). Quanto aos aspectos éticos, o estudo foi aprovado pelo Comitê de Ética em Pesquisa da Fiocruz Minas (CAAE: 34649814.3.0000.5091). Todos os participantes assinaram o termo de consentimento livre e esclarecido específico para entrevista individual e mensuração das medidas físicas.

### IMC

Os desfechos do estudo foram os desvios nutricionais do IMC. O IMC foi calculado dividindo-se o peso do indivíduo (em quilogramas) por sua altura (em metros) ao quadrado (kg/m^2^). As medidas de peso e altura foram aferidas durante a visita domiciliar por meio de protocolos padronizados, que podem ser consultados no *Manual de Entrevista*^17^ e na página de Internet do ELSI-Brasil (https://elsi.cpqrr.fiocruz.br).

Resumidamente, os participantes foram orientados a retirar os sapatos, roupas pesadas e todos os acessórios e objetos dos bolsos. Para aferição do peso, foi utilizada uma balança digital portátil do tipo plataforma, devidamente calibrada (modelo 813, marca Seca; https://www.seca.com). Os participantes subiram na balança, permaneceram com o peso distribuído igualmente em ambos os pés apoiados na plataforma, braços estendidos ao longo do corpo e olhando para uma linha horizontal. Para a aferição da altura, foi utilizado um estadiômetro vertical portátil, marca Nutri-Vida. Os participantes foram orientados a remover penteados, enfeites e prendedores de cabelo. Depois, foram posicionados descalços em pé, de costas para a escala numérica do equipamento, com as pernas e os pés paralelos, peso distribuído em ambos os pés, braços estendidos ao longo do corpo com as palmas das mãos voltadas para o corpo e cabeça alinhada ao plano de Frankfurt para registrar a medida. Ambas as medidas antropométricas foram aferidas duas vezes, sendo utilizadas as médias.

O IMC foi classificado em baixo peso, eutrofia ou excesso de peso, de acordo com o ponto de corte para a idade do participante. Para os adultos (50-59 anos), foram adotados os pontos de corte preconizados pela Organização Mundial da Saúde (OMS) ^5^: “baixo peso” (< 18,5kg/m^2^), “eutrofia” (18,5-24,9kg/m^2^) ou “excesso de peso” (> 24,9kg/m^2^). Participantes idosos (60 anos ou mais) foram classificados nas seguintes categorias: “baixo do peso” (< 22,0kg/m^2^), “eutrofia” (22,0-27,0kg/m^2^) ou “excesso de peso” (> 27,0kg/m^2^), de acordo com os critérios de Lipschitz ^18^, adotados pelo Ministério da Saúde do Brasil ^19^.

### Características sociodemográficas

As características sociodemográficas analisadas foram baseadas em publicações anteriores ^8^^,^^10^: faixa etária em anos (50-59, 60-69, 70-79 ou ≥ 80); cor da pele autorreferida (branca, preta ou outras); estado civil (casado ou solteiro/viúvo/divorciado); escolaridade, considerando os anos completos de estudo (até 8, 9-11 ou ≥ 12); renda domiciliar *per capita* (em tercil); área de residência do domicílio (urbana ou rural); e macrorregião geográfica (Sul, Sudeste, Centro-oeste, Norte ou Nordeste).

### Análises estatísticas

Inicialmente, foi estimada a prevalência de baixo peso, eutrofia e excesso de peso, estratificada por sexo e faixa etária. Posteriormente, foi realizada análise descritiva das características sociodemográficas de acordo com a classificação do IMC para cada sexo, sendo que diferenças nas distribuições foram verificadas por meio do teste de qui-quadrado de Pearson com correção de Rao-Scott, ao nível de significância de 5%. A força da associação entre as características sociodemográficas e os desvios nutricionais do IMC foi estimada por meio de *odds ratio* (OR) brutos e ajustados a seus intervalos de 95% de confiança (IC95%), obtidos por meio de regressão logística multinomial, tendo como referência a categoria “eutrofia”. Foram realizados os ajustes nos modelos de forma sequencial: (1) características sociodemográficas individuais, incluindo faixa etária, cor da pele autorreferida, estado civil, escolaridade e renda domiciliar *per capita* (Modelo 1); (2) características sociodemográficas de localização do domicílio, incluindo área de residência do domicílio e macrorregião geográfica (Modelo 2); e, (3) Modelos 1 e 2 juntos (Modelo totalmente ajustado). O teste de multicolinearidade foi utilizado para testar a correlação entre as variáveis incluídas nos modelos multivariados, por meio do fator de inflação de variância (VIF, do inglês *variance inflation factor*). Como a multicolinearidade não foi evidenciada (VIF = 1,16 para o sexo feminino e VIF = 1,13 para o sexo masculino), todas as variáveis independentes foram mantidas nos modelos totalmente ajustados.

Todas as análises foram realizadas no software Stata/SE, versão 17.0 (https://www.stata.com), utilizando-se o comando *svy* para considerar a complexidade do desenho amostral e o peso individual dos participantes do ELSI-Brasil.

## Resultados

Entre os 9.412 participantes da linha de base do ELSI-Brasil, 8.974 apresentaram informações completas para cálculo do IMC e foram incluídos nesta análise. A maioria dos participantes declarou cor da pele não branca (preta e outras) (57,3%), era casado (63,5%), tinha até 8 anos de escolaridade (73,1%) e residia na área urbana (84,7%). Além disso, 35,1% estavam no 3º tercil da renda domiciliar *per capita* e 47,2% residiam na macrorregião Sudeste (Tabela 1). No geral, as prevalências de baixo peso não diferiram significativamente entre os sexos, sendo observado em 6,9% das mulheres (IC95%: 5,9-8,1) e 8,3% dos homens (IC95%: 7,0-9,9), por sua vez, o excesso de peso foi significativamente maior nas mulheres do que nos homens (64,1%; IC95%: 62,0-66,2 *vs.* 57,3%; IC95%: 54,2-60,3, respectivamente).

**Tabela 1:** Descrição das características sociodemográficas de mulheres e homens mais velhos brasileiros de acordo com a classificação do índice de massa corporal (IMC). *Estudo Longitudinal da Saúde dos Idosos Brasileiros* (ELSI-Brasil, 2015-2016).

Características sociodemográficas	Total (%)	Mulheres				Homens			
		Eutrofia * (%)	Baixo peso * (%)	Excesso de peso * (%)	Valor de p **	Eutrofia * (%)	Baixo peso * (%)	Excesso de peso * (%)	Valor de p **
Cor da pele autorreferida					0,903				0,121
Branca	42,7	28,5	6,9	64,6		32,5	7,3	60,2	
Preta	9,7	28,9	7,7	63,4		35,8	10,6	53,6	
Outras	47,6	29,4	6,5	64,1		35,8	8,8	55,4	
Estado civil					< 0,001				< 0,001
Casado	63,5	27,5	3,9	68,6		32,3	7,3	60,4	
Solteiro/Viúvo/Divorciado	36,5	30,7	10,5	58,8		41,1	11,6	47,3	
Escolaridade (anos)					0,128				< 0,001
Até 8	73,1	29,3	7,3	63,4		36,2	9,5	54,3	
9-11	18,6	26,3	5,6	68,1		28,4	5,8	65,8	
≥ 12	8,3	31,8	6,0	62,2		32,6	3,3	64,1	
Renda domiciliar *per capita* (tercis)					0,679				< 0,001
1º	31,8	28,2	7,5	64,3		36,0	9,8	54,2	
2º	33,1	29,5	7,1	63,4		37,8	10,5	51,7	
3º	35,1	29,2	6,1	64,7		30,1	5,3	64,6	
Área de residência do domicílio					0,005				0,002
Urbana	84,7	28,5	6,3	65,2		33,5	7,5	59,0	
Rural	15,3	31,3	10,2	58,5		39,5	13,2	47,3	
Macrorregião geográfica					< 0,001				0,343
Sul	16,5	25,0	5,5	69,5		32,9	6,3	60,8	
Sudeste	47,2	28,3	5,9	65,8		33,4	8,0	58,6	
Centro-oeste	6,6	31,9	6,7	61,4		34,5	8,1	57,4	
Norte	5,6	29,1	7,1	63,8		30,0	9,5	60,5	
Nordeste	24,1	32,4	9,8	57,8		38,6	10,3	51,1	
Total (n ***)	8.974	1.490	395	3.164		1.409	346	2.170	

* Pontos de corte de classificação do IMC: para adultos (50-59 anos), baixo peso (< 18,5kg/m2), eutrofia (18,5-24,9kg/m2) e excesso de peso (> 24,9kg/m2); para idosos (60 anos ou mais), baixo peso (< 22,0kg/m2), eutrofia (22,0-27,0kg/m2) e excesso de peso (> 27,0kg/m2);

** Valor de p estimado por meio do teste de qui-quadrado de Pearson com correção de Rao-Scott;

*** Número de entrevistados, não incluindo correções de acordo com os parâmetros de amostragem e desenho do estudo.

**Figura 1:**
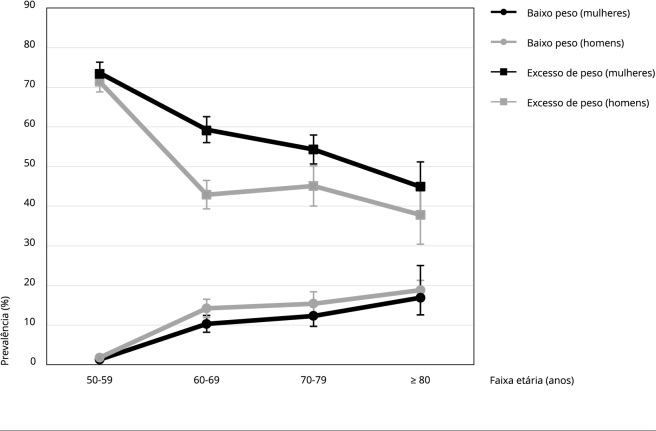
Prevalência de baixo peso e excesso de peso em mulheres e homens mais velhos brasileiros conforme faixa etária. *Estudo Longitudinal da Saúde dos Idosos Brasileiros* (ELSI-Brasil, 2015-2016).

Estratificando as prevalências pelas faixas etárias (Figura 1), observa-se que o baixo peso apresentou relação positiva com a faixa etária em ambos os sexos. As prevalências aos 50-59 anos, 60-69 anos, 70-79 anos e ≥ 80 anos foram de 1,3% (IC95%: 0,8-2,1), 10,3% (IC95%: 8,4-12,6), 12,3% (IC95%: 9,9-15,1) e 16,9% (IC95%: 13,0-21,7) para mulheres e de 1,8% (IC95%: 1,2-2,6), 14,2% (IC95%: 12,0-16,6), 15,4% (IC95%: 12,6-18,6) e 18,8% (IC95%: 13,3-25,8) para homens, respectivamente. Em contrapartida, com o avançar da idade, a prevalência de excesso de peso diminuiu em ambos os sexos - com prevalência de 73,6% (IC95%: 70,8-76,2) e 44,9% (IC95%: 38,8-51,3) nas mulheres de 50-59 anos e ≥ 80 anos, respectivamente; e de 71,4% (IC95%: 68,7-73,9) e 37,8% (IC95%: 30,7-45,5) nos homens de 50-59 anos e ≥ 80 anos, respectivamente.

Em relação às características sociodemográficas, verificou-se diferença estatisticamente significativa (valor de p < 0,05) nas variáveis estado civil, área de residência do domicílio e macrorregião geográfica entre as mulheres e estado civil, escolaridade, renda domiciliar *per capita* e área de residência do domicílio entre os homens (Tabela 1).

As Tabelas 2 e 3 apresentam os resultados da análise bruta e do modelo totalmente ajustado por todas as características sociodemográficas para o sexo feminino e masculino, respectivamente. Considerando-se as mulheres, o baixo peso foi mais presente que a eutrofia em todas as faixas etárias mais avançadas em relação à faixa etária de 50-59 anos, entre as solteiras/viúvas/divorciadas (OR = 1,95; IC95%: 1,42-2,66), assim como entre residentes da área rural (OR = 1,58; IC95%: 1,01-2,49). Ao contrário, a chance de excesso de peso foi inferior à chance de eutrofia em todas as faixas etárias mais avançadas, entre as moradoras da área rural (OR = 0,78; IC95%: 0,62-0,97) e as residentes em todas as macrorregiões geográficas em comparação à Sul. Para os homens, uma maior chance de baixo peso em relação à eutrofia foi observada com o avançar da idade, com gradiente dose-resposta. Já a chance de excesso de peso foi menor em todas as faixas etárias em comparação aos participantes com 50-59 anos e entre solteiros/viúvos/divorciados (OR = 0,58; IC95%: 0,48-0,69). A associação observada entre renda domiciliar *per capita* e IMC evidenciou que os mais ricos apresentaram menor chance de baixo peso (OR = 0,59; IC95%: 0,38-0,90), e maior chance de excesso de peso (OR = 1,52; IC95%: 1,20-1,92).

**Tabela 2:** Associação entre características sociodemográficas e índice de massa corporal (IMC) em mulheres mais velhas brasileiras. *Estudo Longitudinal da Saúde dos Idosos Brasileiros* (ELSI-Brasil, 2015-2016).

Características sociodemográficas	Análise bruta		Modelo totalmente ajustado *	
	Baixo peso **	Excesso de peso **	Baixo peso ** (n = 370 ***)	Excesso de peso ** (n = 3.038 ***)
	OR (IC95%)	OR (IC95%)	OR (IC95%)	OR (IC95%)
Faixa etária (anos)				
50-59	1,00	1,00	1,00	1,00
60-69	6,49 (3,83-10,99)	0,67 (0,54-0,82)	6,36 (3,72-10,90)	0,66 (0,53-0,82)
70-79	7,01 (4,19-11,74)	0,56 (0,45-0,68)	7,02 (4,04-12,20)	0,58 (0,46-0,73)
≥ 80	8,47 (4,97-14,44)	0,40 (0,29-0,55)	6,82 (3,79-12,28)	0,41 (0,28-0,58)
Cor da pele autorreferida				
Branca	1,00	1,00	1,00	1,00
Preta	1,09 (0,72-1,64)	0,97 (0,74-1,26)	0,80 (0,53-1,21)	1,11 (0,86-1,44)
Outras	0,91 (0,67-1,23)	0,96 (0,81-1,14)	0,81 (0,59-1,11)	1,05 (0,88-1,24)
Estado civil				
Casado	1,00	1,00	1,00	1,00
Solteiro/Viúvo/Divorciado	2,39 (1,77-3,21)	0,77 (0,66-0,88)	1,95 (1,42-2,66)	0,88 (0,75-1,03)
Escolaridade (anos)				
Até 8	1,00	1,00	1,00	1,00
9-11	0,85 (0,58-1,25)	1,19 (0,99-1,44)	1,27 (0,88-1,85)	1,03 (0,85-1,25)
≥ 12	0,75 (0,47-1,21)	0,90 (0,68-1,21)	1,14 (0,70-1,88)	0,80 (0,59-1,07)
Renda domiciliar *per capita* (tercis)				
1º	1,00	1,00	1,00	1,00
2º	0,91 (0,63-1,32)	0,94 (0,80-1,11)	0,82 (0,58-1,16)	0,99 (0,83-1,19)
3º	0,78 (0,53-1,15)	0,97 (0,81-1,16)	0,70 (0,45-1,08)	0,97 (0,80-1,17)
Área de residência do domicílio				
Urbana	1,00	1,00	1,00	1,00
Rural	1,47 (0,97-2,25)	0,82 (0,67-1,00)	1,58 (1,01-2,49)	0,78 (0,62-0,97)
Macrorregião geográfica				
Sul	1,00	1,00	1,00	1,00
Sudeste	0,96 (0,56-1,65)	0,84 (0,68-1,03)	1,08 (0,65-1,81)	0,79 (0,63-0,99)
Centro-oeste	0,95 (0,54-1,69)	0,69 (0,52-0,93)	1,13 (0,64-2,01)	0,62 (0,46-0,83)
Norte	1,11 (0,59-2,07)	0,79 (0,59-1,05)	1,30 (0,64-2,61)	0,70 (0,52-0,94)
Nordeste	1,37 (0,81-2,32)	0,64 (0,50-0,83)	1,38 (0,84-2,26)	0,62 (0,47-0,83)

IC95%: intervalo de 95% de confiança; OR: odds ratio, estimados pelo modelo de regressão logística multinomial.

Nota: em negrito, valor de p ≤ 0,05.

* Modelo totalmente ajustado: Modelo 1 (ajustado para características sociodemográficas proximais, incluindo faixa etária, cor da pele autorreferida, estado civil, escolaridade e renda domiciliar per capita) + Modelo 2 (ajustado para características sociodemográficas distais, incluindo área de residência do domicílio e macrorregião geográfica). Categoria de referência: Eutrofia. n total do modelo ajustado = 4.843;

** Pontos de corte de classificação do IMC: para adultos (50-59 anos), baixo peso (< 18,5kg/m2), eutrofia (18,5-24,9kg/m2) e excesso de peso (> 24,9kg/m2); para idosos (60 anos ou mais), baixo peso (< 22,0kg/m2), eutrofia (22,0-27,0kg/m2) e excesso de peso (> 27,0kg/m2);

*** Número de entrevistados, não incluindo correções de acordo com os parâmetros de amostragem e desenho do estudo.

**Tabela 3:** Associação entre características sociodemográficas e índice de massa corporal (IMC) em homens mais velhos brasileiros. *Estudo Longitudinal da Saúde dos Idosos Brasileiros* (ELSI-Brasil, 2015-2016).

Características sociodemográficas	Análise bruta		Modelo totalmente ajustado *	
	Baixo peso **	Excesso de peso **	Baixo peso ** (n = 327 ***)	Excesso de peso ** (n = 2.088 ***)
	OR (IC95%)	OR (IC95%)	OR (IC95%)	OR (IC95%)
Faixa etária (anos)				
50-59	1,00	1,00	1,00	1,00
60-69	5,01 (3,29-7,64)	0,35 (0,28-0,43)	5,50 (3,58-8,45)	0,35 (0,28-0,43)
70-79	5,91 (3,74-9,33)	0,41 (0,32-0,52)	6,30 (3,80-10,45)	0,41 (0,32-0,52)
≥ 80	6,56 (3,58-12,03)	0,30 (0,21-0,42)	7,13 (3,77-13,46)	0,30 (0,21-0,42)
Cor da pele autorreferida				
Branca	1,00	1,00	1,00	1,00
Preta	1,33 (0,83-2,10)	0,81 (0,58-1,12)	1,13 (0,69-1,84)	0,91 (0,63-1,30)
Outras	1,09 (0,79-1,52)	0,83 (0,67-1,03)	1,03 (0,70-1,51)	0,83 (0,69-1,01)
Estado civil				
Casado	1,00	1,00	1,00	1,00
Solteiro/Viúvo/Divorciado	1,24 (0,94-1,63)	0,61 (0,51-0,73)	1,31 (0,97-1,77)	0,58 (0,48-0,69)
Escolaridade (anos)				
Até 8	1,00	1,00	1,00	1,00
9-11	0,78 (0,46-1,33)	1,55 (1,19-2,01)	1,24 (0,71-2,18)	1,08 (0,83-1,41)
≥ 12	0,39 (0,18-0,81)	1,31 (0,88-1,95)	0,52 (0,25-1,08)	1,01 (0,65-1,56)
Renda domiciliar *per capita* (tercis)				
1º	1,00	1,00	1,00	1,00
2º	1,03 (0,71-1,49)	0,99 (0,75-1,11)	0,85 (0,58-1,25)	1,02 (0,82-1,25)
3º	0,65 (0,42-1,00)	1,43 (1,16-1,75)	0,59 (0,38-0,90)	1,52 (1,20-1,92)
Área de residência do domicílio				
Urbana	1,00	1,00	1,00	1,00
Rural	1,49 (1,03-2,15)	0,67 (0,49-0,94)	1,29 (0,90-1,87)	0,77 (0,59-1,01)
Macrorregião geográfica				
Sul	1,00	1,00	1,00	1,00
Sudeste	1,27 (0,78-2,06)	0,95 (0,70-1,29)	1,30 (0,86-1,96)	0,96 (0,79-1,18)
Centro-oeste	1,23 (0,61-2,47)	0,90 (0,50-1,63)	1,19 (0,64-2,22)	0,90 (0,61-1,33)
Norte	1,68 (0,92-3,07)	1,09 (0,64-1,85)	1,42 (0,73-2,76)	1,39 (0,96-2,01)
Nordeste	1,41 (0,84-2,34)	0,72 (0,51-1,00)	1,16 (0,68-1,98)	0,90 (0,68-1,19)

IC95%: intervalo de 95% de confiança; OR: odds ratio, estimados pelo modelo de regressão logística multinomial.

Nota: em negrito, valor de p ≤ 0,05.

* Modelo totalmente ajustado: Modelo 1 (ajustado para características sociodemográficas proximais, incluindo faixa etária, cor da pele autorreferida, estado civil, escolaridade e renda domiciliar per capita) + Modelo 2 (ajustado para características sociodemográficas distais, incluindo área de residência do domicílio e macrorregião geográfica). Categoria de referência: Eutrofia. n total do modelo ajustado = 3.764;

** Pontos de corte de classificação do IMC: para adultos (50-59 anos), baixo peso (< 18,5kg/m2), eutrofia (18,5-24,9kg/m2) e excesso de peso (> 24,9kg/m2); para idosos (60 anos ou mais), baixo peso (< 22,0kg/m2), eutrofia (22,0-27,0kg/m2) e excesso de peso (> 27,0kg/m2);

*** Número de entrevistados, não incluindo correções de acordo com os parâmetros de amostragem e desenho do estudo.

## Discussão

De nosso conhecimento, esse é o primeiro estudo nacionalmente representativo que investigou as características sociodemográficas associadas ao IMC, por sexo, entre adultos mais velhos brasileiros. Os resultados evidenciaram maior prevalência de excesso de peso nas mulheres do que nos homens. Mulheres e homens mais longevos (≥ 80 anos) apresentaram maiores prevalências de baixo peso. Em contrapartida, com o avançar da idade, a prevalência de excesso de peso diminuiu em ambos os sexos. Nas mulheres, o baixo peso foi positivamente associado às faixas etárias mais avançadas, ao estado civil de solteira/viúva/divorciada e à área de residência rural. O excesso de peso foi inversamente associado às faixas etárias mais avançadas, à área rural e a todas as macrorregiões geográficas em comparação à Sul. Para os homens, associações positivas e estatisticamente significativas foram observadas entre o baixo peso e faixa etária. Associações negativas foram observadas entre o excesso de peso e faixa etária, assim como para o estado civil. A renda domiciliar *per capita* associou-se inversamente ao baixo peso e positivamente ao excesso de peso.

É importante considerar a magnitude do baixo peso nos adultos mais velhos brasileiros (6,9% nas mulheres e 8,3% nos homens), ainda que sua prevalência seja bem menor que o excesso de peso e venha apresentando declínio ao longo das últimas décadas ^20^. O baixo peso ainda é bastante frequente nesse segmento populacional, principalmente nos países de baixa e média renda ^8^^,^^10^^,^^21^^,^^22^, contribuindo para o aumento da mortalidade ^23^. Independentemente da fisiopatologia, o baixo peso expressa as disparidades socioeconômicas no acesso e consumo dos alimentos.

Nos últimos anos, inquéritos nacionais têm evidenciado que a prevalência de excesso de peso está em níveis elevados na população brasileira ^6^^,^^7^, e vários países do mundo enfrentam problema semelhante ^24^. A prevalência de excesso de peso é resultado das mudanças globais nos sistemas alimentares, que tornaram os produtos alimentícios ultraprocessados de menor valor nutricional, mais baratos e acessíveis, aliadas à redução da atividade física no trabalho, no transporte, em casa e no lazer, devido ao desenvolvimento tecnológico ^4^. A associação do excesso de peso com maior mortalidade por todas as causas foi amplamente consistente em quatro continentes de acordo com uma metanálise ^25^. Nossos resultados demonstram a necessidade de estratégias voltadas para prevenir e tratar o excesso de peso, mas também o baixo peso, o que está em consonância com os Objetivos de Desenvolvimento Sustentável da Organização das Nações Unidas, que incluem a erradicação da fome e de todas as formas de desnutrição, o alcance da segurança alimentar e melhora da nutrição ^26^.

A prevalência de excesso de peso foi maior entre as mulheres (64,1%) quando comparada aos homens (57,3%), corroborando os achados de outros estudos conduzidos com idosos (60 anos ou mais) de algumas cidades brasileiras ^27^^,^^28^^,^^29^. Essa diferença pode ser explicada pelas mudanças ponderais relacionadas à idade entre os sexos. Por exemplo, nos homens, o processo de envelhecimento é acompanhado por ganho de peso corporal progressivo até cerca de 65 anos e geralmente tende a declinar após essa idade. Por outro lado, nas mulheres, ocorre um efeito platô de ganho de peso corporal por volta dos 75 anos. Isso quer dizer que as mulheres ganham peso e, por conseguinte, aumentam o IMC por um período maior do que os homens ^9^.

Os resultados deste estudo evidenciaram que a idade está fortemente associada aos desvios nutricionais do IMC. Especificamente, foi observado aumento da prevalência de baixo peso e diminuição da prevalência do excesso de peso com o avançar da idade entre adultos mais velhos de ambos os sexos. Esses achados são consistentes com a literatura atual ^10^ e diferem do observado para os adultos desde os 18 anos, em relação ao excesso de peso, para o qual são evidenciadas prevalências crescentes com o aumento da faixa etária, para ambos os sexos, que tendem a diminuir a partir dos 60 anos, em geral ^13^. Esses resultados podem estar associados, em parte, ao viés de sobrevivência, devido ao aumento da mortalidade de idosos obesos antes dos 80 anos ^30^. Outra explicação envolve as modificações da composição corporal com o envelhecimento, que implicam redistribuição da gordura corporal e perda de massa muscular e água corporal ^9^. Cabe destacar, ainda, as alterações fisiopatológicas e psicológicas que afetam diretamente o estado nutricional e fazem parte da complexa rede de desnutrição nos idosos, tais como a anorexia, abuso de álcool, polifarmácia, multimorbidade, sintomas depressivos, ansiedade, solidão e limitações físicas ^31^.

Acredita-se que a redução da desigualdade de renda pode promover melhor qualidade de vida no envelhecimento. A renda é um preditor de um estilo de vida saudável, por favorecer o acesso a serviços e bens materiais ^32^, e da (in)segurança alimentar e qualidade da dieta ^33^. Vale ressaltar que ainda não existe consenso na literatura sobre a relação entre custos e qualidade da alimentação. Alguns estudos indicam que padrões alimentares saudáveis apresentam maior custo ^34^^,^^35^, enquanto outros autores não identificaram essa associação, incluindo evidência nacional ^36^^,^^37^. No entanto, não há controvérsia sobre o aumento da renda influenciar maior consumo de produtos alimentícios ultraprocessados ^38^ e gastos com a alimentação fora do domicílio ^39^, que estão associados a refeições de baixa qualidade nutricional e alto consumo calórico ^40^, contribuindo para o ganho de peso corporal. Neste estudo, os homens mais ricos apresentaram menor chance de baixo peso, bem como maior chance de excesso de peso, corroborando os achados de outro estudo realizado com a população idosa brasileira ^10^. Esses resultados demonstram que o poder aquisitivo protege contra o baixo peso, pelo poder de compra dos alimentos. Contudo, apenas a renda não é garantia de melhoria efetiva na alimentação. Apesar dos mais ricos terem renda para uma alimentação equilibrada e saudável, é necessária a realização de atividades de educação alimentar e nutricional para orientá-los quanto às escolhas alimentares e garantir uma alimentação adequada. Além disso, as políticas públicas devem ser reformuladas para regulamentar a venda e a publicidade dos produtos alimentícios ultraprocessados.

Estudos anteriores demonstram que a escolaridade também está relacionada ao baixo peso e ao excesso de peso em populações adultas ^41^^,^^42^. No entanto, esta análise não suporta essas evidências para adultos mais velhos brasileiros. Um estudo com dados da Vigitel, de 2019, mostrou que a escolaridade influencia diferentemente o IMC dos indivíduos ^8^. As mulheres com menor escolaridade foram mais acometidas pelo baixo peso e pelo excesso de peso. Nos homens, o baixo peso foi mais prevalente entre os menos escolarizados e o excesso de peso foi mais frequente entre os mais escolarizados ^8^. Similarmente, em uma amostra de idosos brasileiros, foram encontradas maiores prevalências de baixo peso quanto menor o nível de escolaridade dos participantes. Contudo, as prevalências de sobrepeso mostraram-se semelhantes entre os níveis educacionais, o que pode ter influenciado a não associação da escolaridade com o IMC ^10^, tal como observado.

Neste estudo, a cor da pele autorreferida não apresentou associação significativa com IMC em ambos os sexos. Outro estudo com idosos (60 anos ou mais) brasileiros identificou menor prevalência de sobrepeso entre aqueles que autodeclararam cor da pele amarela ^10^. Por outro lado, os adultos (≥ 18 anos) negros e de outras etnias minoritárias apresentaram um aumento relativo maior na prevalência de excesso de peso no período de 2006 a 2019 ^43^. De acordo com a literatura, o status socioeconômico ao longo da vida pode explicar algumas das diferenças raciais no IMC ^32^.

Evidenciou-se, também, que as mulheres da área rural apresentaram maior chance de baixo peso e menor chance de excesso de peso, resultados similares aos de outra publicação ^10^. Esses achados podem ser explicados pelas diferenças no estilo de vida, visto que as populações rurais são mais ativas no trabalho e despendem menos tempo vendo televisão ^44^. Além disso, a distribuição geográfica dos desvios nutricionais do IMC revelou menor chance de excesso de peso em mulheres das macrorregiões Sudeste, Centro-oeste, Norte e Nordeste, em comparação às residentes da Sul. Pereira et al. ^10^ encontraram uma tendência similar. Essa espacialização das cinco macrorregiões brasileiras acompanha a heterogeneidade do país. As características ambientais, culturais e socioeconômicas regionais podem repercutir no estilo de vida, hábitos e disponibilidade domiciliar dos alimentos ^45^, o que parece explicar as diferenças para o sexo feminino. De acordo com um relatório conjunto da Organização para Alimentação e Agricultura das Nações Unidas (FAO), com outras organizações, as mulheres da América Latina são mais afetadas pela insegurança alimentar por pertencerem a um recorte mais vulnerável da população ^46^. Por exemplo, as mulheres ganham cerca de 23% menos que os homens e gastam mais horas em trabalho não remunerado ^26^. Garantir a igualdade entre homens e mulheres não é apenas um direito fundamental, mas uma base necessária para promoção dos cuidados em saúde e educação dos filhos, e elementar para o sucesso político e econômico de suas comunidades ^26^^,^^47^.

Neste estudo, as mulheres solteiras/viúvas/divorciadas apresentaram maior chance de baixo peso do que as casadas, enquanto os homens solteiros/viúvos/divorciados apresentaram menor chance de excesso de peso. Dados do estudo *Saúde, Bem-Estar e Envelhecimento* (SABE), conduzido no Município de São Paulo, demonstraram que pessoas casadas de ambos os sexos, com 70 anos ou mais, tinham IMC mais elevado do que as solteiras ^48^. Um estudo realizado com adultos (≥ 30 anos) também comprovou maior chance de sobrepeso e obesidade entre os participantes que viviam com companheiro ^49^. É provável que os adultos mais velhos que vivem sem companheiro estejam mais vulneráveis aos desvios nutricionais do IMC, seja baixo peso ou excesso de peso. O matrimônio ocasiona mudanças no estilo de vida e na assistência de atividades diárias, como a aquisição e preparo dos alimentos, e até nos próprios hábitos alimentares, afetando o peso corporal ^10^^,^^48^^,^^49^ de maneira diferente entre os sexos.

Os pontos fortes deste estudo incluem a representatividade nacional da amostra de adultos mais velhos brasileiros, a utilização de medidas antropométricas diretamente aferidas e o emprego dos pontos de cortes adotados pelo Sistema de Vigilância Alimentar e Nutricional (SISVAN) ^19^ para classificação do IMC dos participantes idosos. Nossos achados ampliam o conhecimento sobre os desvios nutricionais no país segundo as características sociodemográficas e podem orientar as políticas públicas sociais e de nutrição, como a inclusão dos segmentos populacionais mais vulneráveis em programas de prevenção do baixo peso e do excesso de peso e de promoção de hábitos saudáveis. No entanto, este estudo apresenta algumas limitações que precisam ser reconhecidas. A primeira está relacionada à natureza transversal do estudo, que impede inferir relações causais entre as características sociodemográficas e os desvios nutricionais. Outra limitação a ser considerada é o uso do IMC na avaliação dos desvios nutricionais. O IMC não distingue massa magra da massa gorda, portanto, não é capaz de informar sobre a composição corporal dos indivíduos. Infelizmente, medidas de composição corporal não estão disponíveis no ELSI-Brasil. Ainda assim, a avaliação e monitoramento do IMC são essenciais na avaliação nutricional dos adultos mais velhos, por ser um método não invasivo, de baixo custo e de fácil obtenção, apresentando associação com a mortalidade geral e com causas específicas ^23^. Estudos futuros que investiguem outros determinantes dos desvios nutricionais (como fatores comportamentais e condições de saúde) são necessários, principalmente estratificados por sexo e faixa etária, além de estudos longitudinais que avaliem as alterações antropométricas que ocorrem com o envelhecimento.

Apesar das associações entre características sociodemográficas e IMC já terem sido descritas na literatura, os achados deste estudo contemplam uma lacuna ao explorar as diferenças entre os sexos e reiteram a importância de ações de monitoramento do estado nutricional da população adulta mais velha brasileira, considerando as condições contextuais. A influência da escolaridade e da cor da pele autorreferida no IMC necessita ser melhor explorada, visto que não confirmamos as associações descritas anteriormente ^8^^,^^10^^,^^41^^,^^42^. Somente a partir dessa compreensão serão identificados os principais determinantes dos desvios nutricionais. As características sociodemográficas devem ser avaliadas pela equipe multiprofissional, por sua utilidade na triagem de indivíduos em risco de desvios nutricionais do IMC e, portanto, sua ajuda no direcionamento de abordagens preventivas. Sugere-se, ainda, a necessidade de políticas públicas e ações intersetoriais que possam enfrentar as desigualdades sociodemográficas no país e, por conseguinte, auxiliar no enfrentamento dos desvios nutricionais do IMC nesse segmento populacional. Além disso, os programas de alimentação e nutrição, assim como as estratégias de educação alimentar e nutricional, devem levar em consideração as disparidades sociais da população brasileira, e não somente as características individuais, e serem direcionados ao combate tanto do baixo peso, quanto do excesso de peso, pois ambos são característicos da má nutrição e interferem negativamente na qualidade de vida.

## Conclusão

Os resultados deste estudo reforçam as características da transição nutricional, evidenciando a maior prevalência do excesso de peso nas mulheres em relação aos homens e prevalência similar de baixo peso entre os sexos em adultos mais velhos brasileiros. O baixo peso foi mais evidente entre mulheres e homens mais longevos, e a prevalência de excesso de peso tendeu a diminuir com o avançar da idade em ambos os sexos. Além disso, as características sociodemográficas associadas ao IMC diferiram entre os sexos. Faixa etária, estado civil, área de residência do domicílio e macrorregião geográfica foram associados ao IMC entre as mulheres, enquanto faixa etária, estado civil e renda domiciliar per capita foram associados ao IMC entre os homens.
